# Barriers to Accessing Care and Support Services for Older Immigrants and Immigrants with Dementia in Finland: Perspectives of Professional Social and Health Care Providers

**DOI:** 10.1007/s10823-025-09523-2

**Published:** 2025-03-07

**Authors:** Alex Berg, Mervi Issakainen, Kaijus Ervasti, Tero Montonen, Eino Solje, Anna Mäki-Petäjä-Leinonen

**Affiliations:** 1 Faculty of Social Sciences and Business Studies, Department of Law, University of Eastern Finland, Yliopistokatu 2, Aurora B, P.O. Box 111, FI-80101 Joensuu, Finland; 2 Faculty of Social Sciences and Business Studies, Business School, University of Eastern Finland, Microkatu 1 F, Technopolis, P.O. Box 1627, FI-70211 Kuopio, Finland; 3 Faculty of Health Sciences, School of Medicine, University of Eastern Finland, Yliopistonrinne 3, Medistudia, P.O. Box 1627, FI-70211 Kuopio, Finland

**Keywords:** Care and support, Immigrant, Dementia, Legal rights, Access to justice

## Abstract

Older people’s immigration to a different country can place them in a vulnerable situation. Research on the legal rights and access to justice for older immigrants and immigrants with dementia seeking care and support is scarce in Finland. This study addresses this gap in the research and employs semi-structured qualitative interviews with professionals offering services to this target group. Inductive and deductive qualitative content analysis approaches were used to analyze the data. The themes created from the data were analyzed from the perspective of an Elder Law theory, namely, Doron’s *Multidimensional Model of Elder Law.* According to the findings of this study, the major challenges to accessing care and support include linguistic barriers, lack of digital skills, lack of information and knowledge, loneliness, and cultural differences. Some of the strategies that can be adopted to address these challenges include developing culturally sensitive services, dissemination of information about the available services in different languages and employing more bilingual staff.

## Introduction

Older people’s relocation to a different place can add to their stressors (Li et al., 2018 cited Edwards and Rothbard, 1999), placing them in a more vulnerable position. The psychological trauma resulting from relocating to a different country and the accompanying social isolation at the start can increase the risk of cognitive impairment, including the onset of dementia (Chen and Caramelli, 2022). Immigrants with dementia are thus also perceived as vulnerable groups, as many of the immigrants come from war-torn or developing countries, where low levels of education and lack of knowledge about dementia can further add to their challenges (Chen and Caramelli, 2022).

Previous research on older immigrants in Finland is scarce and has consisted only of a few studies. One study found a correlation between lack of linguistic skills and the inability of older Somali immigrants to express their needs to their doctors (Mölsä and Tiilikainen, 2008). A study on Russian-speaking older immigrants showed that the lack of language skills, lack of knowledge about the public welfare system, and limited digital skills impacted older immigrants’ access to digital services (Safarov, 2021). A scoping review on older immigrants in Finland (KC et al., 2023) highlighted the effects of cultural differences, poor language skills, and gender roles on older immigrants’ access to social and healthcare services and inclusion in society. Stereotypes and discrimination are also prevalent in Finnish society, further leading immigrant groups to distancing themselves from the Finnish community (KC et al., 2023). Nonetheless, there is no prior research on immigrants with dementia in Finland.

However, previous research conducted abroad has involved both older immigrants, and immigrants with dementia. When taking older immigrants into account, social isolation was found to lead to loneliness and dependency on others in a study conducted on older immigrants in Canada (Guruge et al., 2021). Lack of information about healthcare services restricted older immigrants’ access to care and support services in the United States (Chung et al., 2018).

A study on immigrants with dementia, on the other hand, showed that limited linguistic skills were associated with incorrect dementia diagnoses in a study conducted on immigrants with dementia in Sweden (Rosendahl et al., 2016) and led to social isolation among immigrants with dementia in Norway (Sagbakken et al., 2020). Traditional family roles where relatives are expected to take care of their older parents affected their access to care and support in a study conducted on immigrants with dementia in Denmark (Nielsen et al., 2020).

Based on the previous findings, it is indicated that older immigrants and immigrants with dementia have unequal access to care and support services. Taking these findings into account, as well as the vulnerable position of these groups of people, and the lack of research on them in Finland, there is a need therefore to investigate the challenges that they can face when they seek care and support services in Finland. Our study fills this gap and addresses an issue that is under researched from a socio-legal perspective. Previous research primarily focuses on the barriers to access to care and support from a social perspective, thus paying little attention to legal aspects. Moreover, as immigration to Finland has constantly increased in recent years (Immigration Department, 2019), the number of immigrants above the age of 50 has increased as well (Wrede et al., 2020). The proportion of older immigrants is further expected to rise considering the increase in the number of older refugees and first-generation immigrants who arrived in Finland in the 1990s (KC et al., 2023). According to recent statistics, there are approximately 32,000 people with foreign backgrounds above the age of 65, where the majority originate from former Soviet Union countries, such as Estonia and Russia. A considerable number of older immigrants in Finland also come from Sweden, Germany, Iraq, Somalia, Iran and Afghanistan. Most older immigrants live in Southern Finland region (Statistics Finland, 2023).

The number of people with dementia is also increasing globally (Chen & Caramelli, 2022). In Finland, it is estimated that 1500 immigrants above the age of 65 have dementia (Monsees et al., 2021). The numbers of immigrants with dementia can be however higher, depending on the likelihood of taking the memory tests. Dementia can also develop at a younger age (Kane & Thomas, 2017).

The increase in immigration to Finland can mean there will be more older immigrants on one hand, and more immigrants with dementia on the other, which can pose more challenges in Finnish society regarding their legal rights and access to justice. From a socio-legal perspective, the concept of ‘access to justice’ has broader meaning, which covers many problems and needs, as well as solutions for them (Cappelletti and Garth, 1981). In our study, we focus on the barriers that older immigrants and immigrants with dementia face regarding their access to social and medical services, and the strategies that can help tackle these barriers. Addressing these challenges can help develop methods that social and healthcare staff can adopt when they offer services to these two groups.

As this article tackles the legal rights and access to justice for older immigrants and immigrants with dementia in Finland, we will answer the following research questions: *(i) What are the challenges older immigrants and immigrants with dementia face when they seek care and support in Finland according to professionals who offer services to them? (ii) What are the strategies that facilitate their access to justice?*

The following section outlines the legal framework concerning their access to care and support services in Finland.

## Legal Framework

According to Section 6 of the Finnish constitution, everyone has a fundamental right to equal treatment. No one must be discriminated against based on their age, origin, language, religion, health, disability, or other reasons. This is in line with Article 2 of the United Nations’ Universal Declaration of Human Rights on equal treatment without discrimination and Article 21 of the European Convention on Human Rights. The European Social Charter, which Finland has signed and ratified, dedicates state parties to ensure the right of everyone to health care (Article 11) and social welfare services (Article 14), as well as ensuring the right of older people to social protection through promoting their active participation in society, independent performance, and access to health care and information about services (Article 23).

These provisions are reflected in the Finnish acts. For example, the Equality Act (1325/2014) aims to promote equality, prevent discrimination, and reinforce the protection of persons who fall victim to discrimination (Section 1). The act prohibits discrimination based on people’s age, origin, citizenship, health condition, disability, language, belief, religion, or any other reason (Section 8). Aspects of equality are not only highlighted in the Equality Act. For example, the Social Welfare Act (1301/2014) also recognizes one of its key principles as ensuring the clients’ right to receive good quality social services and treatment without any discrimination (Section 30). In addition, the act targets promoting the population’s social security, well-being, mental health, equality, and inclusion in society, as well as enhancing their independence (Sections 1, 4 and 7b). The principles set out in the Social Welfare Act apply to people of all ages. Additionally, *the Act on Supporting the Functional Capacity of the Aging Population and on Social and Health Care Services for Older People* (980/2012, henceforward the Elder Care Act) lays down measures on supporting older people’s health, independence, functional capacity, and wellbeing, and providing them with high quality health and social services (Section 1). An older person is identified in this act as “a person whose physical, cognitive, psychological or social functioning has weakened due to diseases or injuries that started, increased or worsened with old age, or due to age-related degeneration” (Section 3).

In Finland, welfare counties are the responsible bodies for the provision of social and health care services. Care services provided to older people in general can be organized as home care, round-the-clock care services, or institutional care. Provision of care in Finland is primarily focused on care at private homes (the Elder Care Act, Section 14).

Home care services are assessed based on the needs of the clients and include home medical care and carrying out activities that strengthen individuals’ functional abilities and performance (Social Welfare Act, Section 19a). Home care services also involve support services for cleaning, catering, clothing, dealing with authorities, and reinforcing social inclusion (Social Welfare Act, Section 19).

The round-the-clock care services involve round-the-clock provision of care services at nursing homes regardless of the time of the day. The services include cleaning, catering, and garment care, as well as arranging activities that enhance functional capacity and social inclusion (Social Welfare Act, Section 21c).

Institutional care involves arranging treatment, care, and rehabilitation in a social care unit either on a short-term or a long-term basis, during the day, at night, or around the clock (Social Welfare Act, Section 22). Institutional care can also be arranged in a health care unit which involves treatment and rehabilitation services (The Health Care Act, 1326/2010, Section 67). Long-term institutional care can only be provided based on medical grounds or reasons related to patients’ safety (Elder Care Act, Section 14a).

Regarding persons with disabilities, their inclusion and participation in society on an equal basis with others are some of the objectives stipulated in Section 1 of the Disability Services Act (675/2023). The act defines a disabled person as a person who has a long-term or permanent physical, cognitive, psychological, or social limitation (Section 2). The Disability Services Act guarantees more services and support compared to other acts, such as transport service (Section 28), personal assistance (Section 9) and training to learn new skills that support independent living (Section 7). Finland had also a national program that aimed at promoting brain health and early detection and treatment of cognitive disorders, such as dementia. Nonetheless, since 2021, these goals have been instead moved to a broader national aging program (Terveyden ja hyvinvoinnin laitos, 2023).

In Finland, access to social and health care services in general requires residence in a welfare county according to the Act on the Organization of Social and Health Care (612/2021, Section 2). Concerning foreigners moving from abroad, registration of a place of residence requires holding a continuous (Type A) or a permanent (Type P) residence permit in Finland as specified in Section 4 of the Home Municipality Act (201/1994). If the person has a temporary residence permit, i.e. Type B permit, e.g., for study exchange, the permit should be given for at least for one year with the intention of staying in Finland permanently (Section 4). Immigrants’ right to access social and health care services is thus only restricted when their stay in Finland is perceived as temporary.

The Finnish acts, hence, do not distinguish between a citizen and a non-citizen regarding access to services. Our immigrant groups’ residence in Finland is usually of a permanent nature. The principles set in the Finnish acts apply to them on par with Finnish citizens residing permanently in Finland.

### A Multidimensional Model of Elder Law

This empirical research employs Doron’s (2009) *Multidimensional Model of Elder Law* in driving the analysis and contributing knowledge to Elder Law theories. Doron’s (2009) model involves five dimensions, which address multiple aspects of Elder Law. The five dimensions are:* the Legal Principles Dimension, the Protective Dimension, the Preventive Dimension, the Empowerment Dimension, and the Supportive Dimension.*

The Legal Principles Dimension outlines the basic legal principles in society that ensure the protection of human rights. The protection of civil rights applies to all members of society, regardless of their age. An example of this is the principle of equality. Everyone is equal before the law when it comes to their civil rights and protection against discrimination. Older people can be discriminated against based on their age. Equality and protection against discrimination based on age, for example, are some of the “central legal aspects” of most modern societies, as Doron points out. Different laws can involve equality and protection against discrimination as general principles. Doron clarifies that this has provided a basis for the protection for the older population against discrimination on the same footing as other groups in times or places where a specific law that targets the protection of older people’s rights did not exist. When the legal principles cover all population groups, it proves “advantageous” for older people as these principles consider older people “as an integral and equal part of society” (Doron, 2009, p. 61). This conforms with the concept of “universalism” which values the development of policies that promote people’s active participation in society regardless of their age (Surtees, 2009), which in turn can also reinforce older people’s legal rights and inclusion.

Moreover, older people’s “personal” and “social vulnerability” can make them prone to abuse and exploitation by others (Hall, 2009). With this taken into account, general principles alone can fail to fully protect their rights and address their needs (Doron, 2009). The Protective Dimension is a specific legal dimension that can provide them with better protection, such as protection against abuse and exploitation. The laws concerned with protecting older people and vulnerable groups from financial exploitation, sexual abuse, physical abuse, mental abuse, and neglect are necessary to address their specific needs. An example of protective legal measures against abuse is to oblige state employees, especially social and healthcare professionals, to report and intervene in cases of neglect and abuse of an older or vulnerable person in the family. Abuse, exploitation and negligence can negatively impact the provision of the necessary care and support for older people and vulnerable groups (Doron, 2009).

Furthermore, the aspect of autonomy is also a core value to address in the promotion of older people’s rights (Hall, 2009). Doron’s Preventive Dimension aims at reinforcing older people’s abilities to decide for themselves independently (Doron, 2009). This dimension focuses therefore on establishing legal planning tools that allow older people to plan their lives, and influence decisions later in their lives when they lose the physical and mental capacity to make their own decisions. The legal planning tools can cover matters related to their finances and medical treatments. The Preventive Dimension underlines the importance of protecting older people’s independence, self-respect, as well as sustaining their autonomy (Doron, 2009). The process of planning is flexible in its essence to address their personal needs. In other words, older people’s values, as well as their cultural and ethnic differences are taken into account in the legal planning tools (Doron, 2009).

In addition, the Empowerment Dimension focuses on the importance of providing legal support that aims to empower older people. Some examples of empowering older people can include “providing legal information, advocacy and representation” which “will allow older people to be more aware of their rights, enable them to exercise existing legal rights, and eventually enforce greater respect for older people’s rights” (Doron, 2009, p. 69). However, developing specific policies that aim to improve older people’s lives, wellbeing, and financial situation is not sufficient when older people lack the necessary knowledge and awareness about their legal rights, why they are in place, or how to seek them. It is necessary, therefore, to empower older people to improve their lives as active members of society, which can in turn fulfill the purposes of the other dimensions (Doron, 2009).

Finally, families and communities can play an important role in promoting the rights of older people. The Supportive Dimension takes into account the informal social support that older people receive. Doron argues that legal protection alone is not sufficient to ensure the rights of older people and address their needs. Doron recognizes research findings which concluded that there was a “correlation between quality of life, health, and the ability to age in a respectful manner and the involvement of informal, social support networks” (Doron, 2009, p. 65). For example, in cases where older people had supportive family members “who felt obliged to provide for and support them,” there was less likelihood of sickness and need to place them in care institutions (Doron, 2009, p. 65 cited Kane and Penrod, 1995). Providing legal support to social support networks can in turn promote the rights of older people and their families (Doron, 2009). For example, legal support can involve providing informal carers with financial support as compensation for their caring obligations (Doron, 2009). However, the provision of informal support may not necessarily work to the advantage of the older person. This is because the issue of elder abuse is on the rise, especially from family member caregivers (Morgan, 2009).

In this study, the principles of equality, empowerment, autonomy, and protection against discrimination and abuse in Finland are examined from the perspective of Doron’s model. The study also addresses the role of families and communities in promoting the immigrant groups’ access to justice. Doron’s model involves therefore both the impact of the legal instruments and the social networks on their experiences. While the model addresses the rights of older people as vulnerable groups, the use of this theory in this study that investigates the legal rights and access to justice for older immigrants, and immigrants with dementia, is based on our perception that both of these groups are also vulnerable groups.

### Data and Methods

The research employed semi-structured qualitative interviews with different professionals working as directors of non-governmental organizations (NGOs), medical doctors, social workers, and interpreters. The professionals (*n* = 16) offered services to older immigrants and immigrants with dementia. The professionals had firsthand experience of dealing with people from different cultural backgrounds and offered services directly to people from Russia, Estonia, Somalia, Afghanistan, and the Middle East and North Africa Region. Some of the professionals had foreign backgrounds. Their knowledge on the topic was therefore deemed invaluable for exploring the immigrant groups’ experiences. The professionals were sought by contacting different NGOs and welfare counties and were chosen based on their experience with providing services to older immigrants and immigrants with dementia. Invitations to participate in the research were sent to all regions in Finland, but only participants from the Southern Finland region contacted us. The region of Southern Finland is the most populated region in Finland, and includes big cities like Helsinki, Vantaa and Espoo. We believe that we only received participants from Southern Finland because it has the highest number of immigrant populations, and many NGOs that offer services to older immigrants and immigrants with dementia operate in this region. The professionals consisted of 14 women and two men, who worked in the public and the third sectors. Some of the professionals offered services exclusively to older immigrants and immigrants with dementia.

Even though the research involved a small number of participants, the same answers were repeated in different interviews, indicating data saturation.

The interviews consisted of open-ended questions where the professionals could provide their answers freely. All the interviews were conducted by the first author, who moved to Finland to conduct this research, and had no personal experience or prior knowledge about the experiences of older immigrants and immigrants with dementia. The first author is a man from a Middle Eastern background and a native speaker of Arabic. While he is fluent in English, his Finnish skills are limited, which is why some of the interviews were conducted in English (*n* = 10) and others in Arabic (*n* = 6). Interpretation services were not needed, as the participants spoke English fluently as well, while others had Arabic as their native language. The interviews were audio recorded and consisted of eight individual interviews and three group interviews, of which two group interviews involved two professionals each, and one group interview involved four professionals. Group interviews involved participants who worked at the same organization and preferred to discuss and share their experiences together. While seven interviews lasted between 30 minutes to 67 minutes, four of the individual interviews were short and lasted for 15 minutes (*n* = 1), 16 minutes (*n* = 1), and 22 minutes (*n* = 2). The data collection process took 13 months from May 2023 to June 2024.

The interview guide was developed based on the study’s interest in addressing the challenges to accessing care and support for older immigrants and immigrants with dementia. For example, the professionals were asked about their clients’ ability to seek social and medical services on their own, and whether they feel they have equal access to services in Finland.

To answer the research questions, a qualitative content analysis approach was adopted as a method for its flexibility, enabling us to generate themes from the interview transcripts, and to analyze the created themes from the perspective of a theoretical framework Table 1.

**Table 1:** The participants' details

Participants (*N* = 16)	Area	Recipients of Services
Social Worker (*N* = 2)	Southern Finland	Older Immigrants and immigrants with dementia from Russia, Estonia, Somalia, Afghanistan, and the Middle East and North Africa region
Diversity Work Expert (*N* = 1)		
Project Coordinator (*N* = 1)		
Medical Doctor (*N* = 1)		
Director of an NGO (*N* = 1)		
Intercultural Services Coordinator (*N* = 1)		
Chief Executive Officer of an NGO (*N* = 1)		
Interpreter (*N* = 2)		
Volunteering Hotline Service Agent (*N* = 1)		
Care Assistant (*N* = 1)		
Services Consultant (*N* = 1)		
Development Manager (*N* = 1)		
Activity Organizer (*N* = 2)		

### Analysis

The qualitative data from the semi-structured interviews was analyzed following a combination of inductive and deductive qualitative content analysis approaches (Hsieh and Shannon, 2005). The audio records of the interviews conducted in English were first transcribed verbatim by a transcription service. Considering the interviews conducted in Arabic, the interviews were transcribed and translated directly to English by the first author. The dataset was afterwards analyzed manually.

At the beginning, the first author read through the interview transcripts to get an insight into the whole text. The interview transcripts were then read thoroughly to generate initial codes from the data. The generated codes were then labeled and grouped into categories based on common characteristics shared between the codes. Based on these categories, the final themes were created.

The analysis afterwards was theory oriented as the themes were analyzed from the perspective of an Elder Law theory, namely, Doron’s (2009) *Multidimensional Model of Elder Law.* Each theme triggered different dimensions from Doron’s model. The analysis from this theoretical lens involved investigating whether Finnish laws and their application in practice are in line with Doron’s dimensions regarding the promotion and protection of older people’s rights. Not only did Doron’s dimensions involve the legal aspects, but also the role of family members and communities in promoting older people’s rights. The goal of the theory-oriented analysis is not only to provide supporting or non-supporting evidence for the theory, but also to extend knowledge derived from this research into theories addressing Elder Law.

## Results

The following table shows the frequency of the occurring themes among the participants. All the participants’ names have been pseudonymized. The table also highlights which dimensions of Doron’s model were involved, and consequently the Finnish regulations concerning these dimensions Table 2.

**Table 2:** The frequency of the participants' inputs, Doron's dimensions, and Finnish laws within each theme

Theme	Participant(s)	Doron’s Model	Finnish Laws
*The Linguistic Barrier*	All participants	*Legal Principles Dimension; Empowerment Dimension*	The Equality Act; Social Care Customers Act; Patient Act; the Act on the Organization of Social and Healthcare; the Act on the Integration of Immigrants and Reception of Asylum Seekers; the Act on the Promotion of Immigrant Integration
*Lack of Digital Skills*	Hanna; Elisa; Tarja; Nour	*Empowerment Dimension*	Social Welfare Act; Disability Services Act
*Lack of Information about Rights*	Elisa; Sara; Sirpa; Lana; Suvi; Laura; Heidi; Amjad; Shams; Rama; Anas; Tarja; Nour	*Empowerment Dimension*	The Act on the Promotion of Immigrant Integration; the Home Municipality Act; the Act on the Organization of Social and Healthcare
*Lack of Expertise among Service Providers*	Hanna; Elisa; Sara; Sirpa; Heidi; Amjad; Shams; Tarja; Rawan	*Legal Principles Dimension*	Social Welfare Act; Elder Care Act; the Equality Act
*Lack of Family and Friends*	Hanna; Elisa; Heidi; Amjad; Rama; Anas	*Supportive Dimension*	Social Welfare Act
*Abuse*	Elisa; Sara; Heidi; Anas; Tarja	*Supportive Dimension; Protective Dimension*	Social Welfare Act
*Cultural Expectations and Conceptions*	Hanna; Elisa; Sara; Suvi; Heidi; Laura; Tarja	*Preventive Dimension; Supportive Dimension; Empowerment Dimension*	Elder Care Act; Social Welfare Act; the Act on Support for Informal Care; Health Care Act

### The Linguistic Barrier

According to the professionals, the linguistic barrier was highlighted as the most prominent obstacle to accessing care and support services in Finland. Immigrant groups are dependent on interpreters, family members, or help from friends and other immigrants. The professionals pointed out that many lacked English language skills as well. Reliance on others for communication delayed, and in some cases restricted their access to needed services. Limited Finnish skills also resulted in service providers ignoring them, as Sirpa, who is a project coordinator at an NGO, noted:

When you go to health services, all of sudden you hear that the person does not speak Finnish … then they’re all of a sudden like, okay, this is not my business … because this person cannot speak … the same language…

It can be argued, however, that the use of interpretation services can help overcome the linguistic barrier. In Finland, clients have a right to an interpreter if the social and healthcare professionals do not know their language according to the Social Care Customers Act (812/2000, Section 5), the Patient Act (785/1992, Section 5), and the Act on the Organization of Social and Health Care (Section 5). This is laid down to ensure equal access to services without discrimination. Doron’s Legal Principles Dimension views equal access to legal rights for everyone without discrimination as crucial to protecting older people’s legal rights, as they can be discriminated against based on their age (Doron, 2009). In this study, immigrants can be discriminated against based on their age, origin, language, and health status. Section 1 of the Equality Act, Section 30 of the Social Welfare Act, and Section 3 of the Patient Act are examples of protecting the population against discrimination, which Doron (2009) argues as “central legal aspects” of many modern societies. However, reliance on interpreters may not in all cases help them overcome their barriers to communication. This is because a non-specialized interpreter might be booked, leading to essential information not being revealed, as the professionals stated, which can still hinder them from enjoying equal and adequate access to services.

Would living in the Finland for a long time and learning Finnish then overcome the language barrier obstacle? Section 7 of the Act on the Integration of Immigrants and Reception of Asylum Seekers (493/1999) and Section 11 of the Act on the Promotion of Immigrant Integration (1386/2010) lay down provisions regarding promoting the integration of immigrants in society. The integration plan involves enhancing immigrants’ linguistic skills in Finnish or Swedish. This goes hand in hand with Doron’s Empowerment Dimension which underlines the need to empower older people with knowledge and the capacity to become active members of society. Empowering older people with knowledge can increase their chances of accessing their legal rights guaranteed in other laws (Doron, 2009). When it comes to immigrants, we can argue that if they learn Finnish or Swedish, it can become easier for them to seek help on their own. However, it can be more difficult for older immigrants to learn the language. While tackling the language barrier can increase their chances of approaching social and healthcare service providers, the ability to speak Finnish may still not necessarily facilitate their access to justice. According to the professionals, the service system in Finland is designed for the ethnic Finns and does not address the needs of people from different cultural backgrounds.

Immigrants with dementia, on the other hand, are at a higher risk of restricted access to justice. Heidi, a medical doctor, underlined that they might lose what they had learnt at a later stage:

… Even sometimes when these people have learned Finnish or speak quite good English … when you get dementia, you sometimes lose your second or third language, and you, in a way, [resort] only to your native tongue and we don’t have of course daycare centers and specially we don’t have permanent care in Finland…

It can be argued as a result that this can place immigrants with dementia in a more critical situation compared to older people in general, and older immigrants in particular. This is because if they lose the language they learned in the host country, not only may they lose the ability to access care and support services, but they may also become isolated from their surroundings and suffer from loneliness, especially if they lived at care institutions.

### Lack of Digital Skills

With Finland being one of the most digital countries in the world (Kippo, 2023), lacking digital skills can pose an obstacle to seeking care and support with the increase in the supply of digital social and healthcare services. According to the professionals, lacking digital skills in combination with not knowing the language can further cripple access to digital services. The inability to use the digital services means that clients cannot navigate the welfare system and access the needed services. The issue is that because there is an increased likelihood of limited Finnish skills, they may not be able to access the services following the conventional methods either, for example by calling the doctor and booking an appointment over the phone. Recently, members of NGOs cannot call and book appointments on behalf of the clients anymore either, as Hanna, who is a social worker, explained:

Because the elderly, if they don’t have … digital [skills], they don’t have the language, they have no way to get the service … Before, we [could] … call to book appointment in the healthcare center. But … now we cannot do it anymore because there is [a] new guidance … [in] the Helsinki city that [the] person need[s] to give [an] official paper authorized for [another] person to do that kind of stuff.

As mentioned, Doron’s Empowerment Dimension emphasizes the importance of empowering older people with the skills needed to improve their capacity to participate in society on par with others. However, while the Finnish Acts guarantee immigrants the right to learn Finnish or Swedish, they do not specify provisions for enhancing their digital skills. Therefore, when it comes to empowering older people with digital skills, we cannot argue that Finnish laws are in line with Doron’s Empowerment Dimension. It is crucial to empower older people with digital skills in a digital country like Finland.

According to the Ombudsman for older people, older people do not have equal opportunities to learn and maintain digital skills, and only 25% of people over the age 79 consider their ability to use the Internet as good (Topo et al., 2024). However, the Social Welfare Act aims to promote people’s independent performance (Section 4), as well as equality and inclusion in society (Section 1). Moreover, according to Section 7 of the Disability Services Act, a disabled person can receive training to learn a skill or improve an existing one which can reinforce their independence. While we can argue that equality and independent performance can be promoted by improving people’s digital skills, the laws need to precisely lay down measures that aim at equipping older people and people with disabilities with the necessary digital skills to navigate the Finnish welfare system.

Therefore, while lack of digital skills can hinder anyone, regardless of age and background, from accessing care and support services, the unique situation of our target group can even make it more challenging for them. It can be argued that even if they had enough digital skills to use mobile phones and computers, the language barrier mentioned above can pose a challenge for them to find the services they need. The use of functions such as automatic translation of webpages may not necessarily enable them to navigate the services and use them. This is because, for example, some people might come from countries that do not extensively use digital services like Finland, which means that they may be unfamiliar with these types of services system and hence find difficulties in seeking the services they need through digital means.

### Lack of Information about Rights

The professionals highlighted that some immigrants do not know about their rights in Finland. It would be impossible for them to know which sections of the laws that guarantee their rights to certain services when even Finnish people do not know about their rights and such laws. The immigrant groups are also more likely to isolate themselves and become less aware of their rights, as Lana, who works as an interpreter, highlighted:

The problem is that they can only know about their rights through the organizations. But if they didn’t go to organizations, or anywhere, they would not be able to know about their rights or anything else. That is the issue. They sit at home. No one will go to their homes and bring them news and tell them where they can get the services.

Amjad, who is an immigrant working as a hotline service agent, stressed that not only do older immigrants not know about their rights, but they are also unaware of their obligations:

They live in a state of isolation. They do not know about the news. They do not know about their rights. At the same time, they do not know about their obligations either . . . They do not know how to ask for something if they need it. They do not know if they are entitled to that right or not.

Doron’s Empowerment Dimension emphasizes the need to empower older people with knowledge about their legal rights so that they can utilize them. If we take the Finnish laws into account, Section 7 of the Act on the Promotion of Immigrant Integration requires authorities to inform immigrants about their rights and duties when they receive residency rights in Finland. This refers to a booklet called *Welcome to Finland*, which was issued by the Ministry of Economic Affairs and Employment of Finland (2022). The booklet is available in many languages. Pages (25) and (52) of the booklet include information about one’s right to social security and healthcare, respectively. The information in the booklet is based on the Home Municipality Act (Section 4) and the Act on the Organization of Social and Health Care (Section 2). The Finnish laws, therefore, aim at empowering older people with information about their rights, if we take Doron’s Empowerment Dimension into account. However, even though the provision of information is stipulated by the law, it is evident that many are not aware of their rights in Finland. Nour, who works as an activity organizer for older immigrants, mentioned that:

Regarding the laws, when you arrive in the country, they give you papers in a language you know, “Here you go. You can read them.” But will the person read these laws? Can this person understand them? . . . . Organizations operating with the same language of the client can help you get the information differently, rather than just a literal translation, because there are laws and information that can be difficult for me to understand if they are translated literally . . . So there is an issue with understanding the information, and how to respond to these information.

Nonetheless, as we mentioned that even Finnish people may not be aware of their rights, the situation of older immigrants and immigrants with dementia can further restrict their chances of accessing information about their rights due to several factors. For example, they may lack necessary language skills to be able to find information and get acquainted with their rights. The language barrier can additionally restrict their abilities to utilize the digital services which are essential to find and access information about their rights.

Furthermore, the lack of social interactions with other people can also make it more challenging for them to know about their rights through social networks and communities. Provision of information about rights and where to seek services can be especially crucial for older immigrants and immigrants with dementia, as according to our professionals, such information can be difficult for them to comprehend because of their vulnerable situation. This is because many of the care and support services provided in Finland are not available in the target group’s home countries, which may make them unaware of the services and rights they are entitled to.

### Lack of Expertise among Service Providers

The professionals in this study highlighted that new employees in the social and healthcare sectors do not get the chance to learn enough about their new work. Many of the decisions are also taken by employees who are either still studying or who have just graduated. Some of these decisions are consequently not based on experiences with dealing with people with foreign backgrounds. Decisions can be, however, based on assumptions and stereotypes about ethnic minorities, according to our professionals. They also added that assumptions and stereotypes about people from ethnic minority groups can make service providers hesitant to offer services to them, which can hinder them from receiving the necessary services on an equal basis with other people and can result in a structural discrimination against them. The laws that protect everyone from discrimination based on their age can in turn protect older people’s rights, as Doron (2009) argues in the Legal Principles Dimension. In our study, the provision of services without discrimination based on age and background is stipulated in Finnish law, which can work for the benefit of immigrants in this case. For example, the Equality Act prohibits discrimination based on age and origin (Section 8). Moreover, according to Section 30 of the Social Welfare Act, everyone has the right to receive good quality social and health care services without any discrimination.

Nevertheless, the provision of adequate and high-quality services to people from different ethnic backgrounds can make it even more challenging for both new and experienced service providers, as our participants underlined. Tarja, who is a development manager, attributes the lack of expertise among professionals to Finland’s exposure to a new, unfamiliar challenge:

… We haven’t had that yet, and you know, when in the 1990s, Somalis and Iraqis started to come here, maybe some of them were like 30, obviously there were probably a few elder people here and there, but now we’re finally seeing people are starting to reach their 60s, people are starting to reach older age, obviously more older people are also coming to Finland. So how do we deal with those challenges of people potentially being lost in the system for 20 years, and all of a sudden they’ve got Alzheimer’s and they need to go to a nursing home and you know, how do we make sure that we know where these people are, what kind of treatment they need, and how do we provide that for them.

Section 10 of the Elder Care Act requires service providers to have the necessary diverse expertise to ensure the adequate organization and provision of needed social and healthcare services for older people. The situation in practice is different, however, considering our target group. This is because there may be uncertainty among service providers, regarding for example whether this kind of service or treatment is acceptable in their culture or not. The Finnish laws therefore conform with Doron’s Legal Principles Dimension regarding access to legal rights for older people without discrimination. However, when the laws are applied in practice, the provision of good quality services is jeopardized when the services are offered to immigrant groups. As one of the interviewees explains:

… We have often situations where someone comes and says, I need help, I need some appointment somewhere … we book them appointment, we send them there. Then after six months, it's still not solved when we help them … where have they been? They've been 20 times to that place. Why [is it] not solved? (Elisa, a diversity work expert)

As a result, it is evident that service providers do not have the means to deal with people from diverse backgrounds. In the case of older immigrants and immigrants with dementia, the lack of expertise can be argued to be a significant issue as these vulnerable groups’ access to care and support services may be delayed or hindered due to the lack of understanding of their specific needs, potentially affecting their physical and mental wellbeing. Providing them with inadequate services can leave them with the belief that they were discriminated against, which may impact their trust in the Finnish social and health care system, and consequently restrict their access to care and support.

### Lack of Family and Friends

The professionals emphasized that many immigrants have no one to help them. They are completely alone and live on their own. One of the NGOs that our professionals work at estimated that around 30% of their clients do not have friends or family to help with the language or digital services. Loneliness can also impact their ability to integrate and become part of the Finnish society.

Their [life] is a series of difficulties. They came here as old people. Those who came with a family, their situation is a bit better, than those who came alone. Those who came with their family, they have children or spouses. They can to some extent rely on each other. However, the old woman or the old man who came alone, it is almost impossible for them to learn the language because they are occupied with other stuff than the language …. [their service needs] are not met for the most part … I know an old person whose English is zero, while his Finnish is under the zero. But he has kids whose Finnish is very good, so his situation is very smooth. But what about those who live alone or those who are divorced and cannot contact their children or do not have children? (Amjad).

This highlights the importance of the role of social supportive networks in the promotion of the rights of older people, as Doron (2009) argues in the Supportive Dimension. Finnish regulations lay down measures that support the participation of close relatives and other people in taking care of individuals who are unable to manage their lives and fulfill their service needs on their own (the Social Welfare Act, Section 43). However, a lack of relatives and friends in some cases undermined their ability to receive the help they need.

Anas, who works as a services consultant, emphasized that while immigrants in general might have the same needs as older Finnish people when it comes to having friends and relatives, there are factors that can increase their risk of loneliness:

… Older immigrants are different from Finnish older people when it comes to loneliness. Older immigrants’ social networks are not that optimal. While when you are in your home country, you would have relatives, friends, you know … But when they come to Finland, the main issue that they face is loneliness, and lacking social networks …. Here, because of loneliness, the linguistic differences, and the cultural differences, the older immigrants are always in need of help from someone else to guide them.

Not having anyone to help also meant that they would try to seek help from the wrong places, elevating the risk of exploitation, as the professionals warned. Therefore, not only can lack of family and friends pose a challenge to accessing care and support, but it can also result in loneliness, dependency, and increase the risk of exploitation. Moreover, Rama, who is a care assistant at a care institution, explained that not having someone to help can place older immigrants in a weaker position when it comes to getting their rights:

Those who have families, of course their families help them. But those who have no one, they only have God …. Even those who expose themselves as strong, they are actually having it bad, especially those who have no one … Because those who have family, and their spouses or children are responsible for taking care of them, they would appear strong. Why is that? Because when they want to complain about the contact persons to the state for not taking care of them, the contact person will fear that and do what the client would ask for. In this case, there is someone to follow up with things. Not those appointed by the state.

Thus, while older Finnish people or Finnish people with dementia might face similar issues resulting from loneliness, the issue with older immigrants and immigrants with dementia is that they might be more prone to experiencing loneliness as a result of moving between countries. This can be especially the case for some older immigrants who moved to Finland many years ago. According to a study on transnational relationships of older immigrants, ties and connections with home countries tend to break over time, which can in turn exacerbate loneliness and exclusion among older immigrants if they were unable to establish new social networks in their host country (Heikkinen & Lumme-Sandt, 2013).

### Abuse

The professionals in this study reported cases of both financial and physical abuse within the same family households. They mentioned that caregivers are more likely to financially abuse their vulnerable relatives. Abuse can therefore have detrimental effects on the vulnerable people’s wellbeing. In these cases, abusive caregivers cannot be identified as “supportive” family members if we take Doron’s (2009) Supportive Dimension into account. However, not only does financial abuse occur within family households, but friends can also financially exploit vulnerable people, as Anas and Tarja explained. Abuse can therefore undermine the role of all informal social support networks in promoting vulnerable people’s wellbeing.

Physical abuse, on the other hand, is mainly exhibited by immigrants with dementia towards their caregivers, as NGO director Sara explained. However, Elisa pointed out that traditional arranged marriages can further intensify the instances of abuse among immigrant families:

… If it's the male who is being taken care of [by] his spouse…. they didn't perhaps even have a love marriage. It was just like decided long time ago, you're going to get married and they cannot handle each other. They don't like each other. Or she has tolerated [him] for a very long time. And now this person has dementia. He's abusive … but he cannot manage his life, he cannot remember himself … and … [his] wife … is forced to take care of him, handle his abuse…

It can be argued therefore that physical abuse towards caregivers can negatively impact their ability to provide care to their relatives, placing immigrants with dementia at a higher risk of restricted access to care and support as abusive relationships between family members from immigrant backgrounds can be immune to external interventions. This is because service providers are hesitant to intervene when the case concerns immigrants due to fear*.* Fear involves both service providers and immigrant groups, according to our professionals.

On the one hand, social care staff did not intervene in cases of abuse between immigrant families. The reason behind this is that they feared being accused of discrimination. If the situation involved Finnish couples or families, the service providers would take action immediately or otherwise there would be a scandal, as one of the interviewees explained:

… If this would be a Finnish couple, this would be a scandal, this would be in the news, this would be abuse and this would not be tolerated. And they would even go against the spouse and against the children to secure both these older people. But because it is [a] migrant couple and there is religion and everything, I've had throughout the years, I had a situation when one of their social workers actually cried [on] the phone to me and said we don't know what to do. (Elisa)

Doron’s Protective Dimension addresses the importance of protecting older people from abuse by ensuring that professionals report and take action against abuse (Doron, 2009). Section 11 of the Social Welfare Act stipulates that victims of violence, abuse and exploitation have the right to receive support. Therefore, Finnish laws guarantee the provision of support to victims of abuse, and they are in line with Doron’s Protective Dimension. However, professionals do not comply with the Finnish laws when the victims of abuse are immigrants. Fear of being accused of discrimination is evidently more important for the service providers than abiding by the law and complying with their obligations and responsibilities towards their clients.

On the other hand, however, the relatives of the aggressive family members feared that if they approached the authorities and sought help as victims of abuse, they would lose their family unit. Our professionals warned that this caused the victims of abuse to remain silent about their experience and retain the status quo.

### Cultural Expectations and Conceptions

Cultural differences play an important role in immigrants’ access to care and support due to different approaches to taking care of family members in different cultures, as our professionals highlighted. For example, the professionals stated that it is acceptable for some children from immigrant backgrounds to decide things for their older parents. The professionals referred also to cases where children would not accept treatment from official healthcare professionals, stating that they would rather take care of their parents themselves.

Doron’s (2009) Preventive Dimension aims at strengthening older people’s autonomy. The Preventive Dimension takes into account legal planning tools that address the special personal needs of older people, such as their values and their ethnic and cultural differences (Doron, 2009). According to Section 13 of the Elder Care Act, when assessing the service needs of older people, the services must support the older person’s health, independent performance, and participation in making decisions. Respecting the customers’ autonomy and right to self-determination is also guaranteed in Section 36 of the Social Welfare Act when assessing the service needs of the population regardless of their age. The Finnish laws in this regard do not conflict with Doron’s Preventive Dimension. However, cultural differences can impact the immigrants’ self-determination and clash with laws that promote autonomy. Consequently, while legal planning tools should accord with older people’s individual values and cultural differences to maintain and respect their personal autonomy, Doron’s Preventive Dimension does not address the potential impact of these values and differences in conversely restricting their right to autonomy.

It is important to note, however, that as children usually have better Finnish skills, they feel they have more power to make decisions for their parents, as Sara emphasized:

… The children actually they are not the carers per se, but they still make the decisions. And they are trusted by the professionals because they may speak better Finnish or English, and they can sort of help out the professionals in a way. But then the rights of the older persons are not met…

Moreover, the professionals underlined that it is also common in some cultures for relatives to hide dementia from their older family members either out of respect or to protect them from knowing about a serious disease. Medical doctor Heidi explained that it used to be like that with cancer in Finland.

Sometimes we find out that people can be quite … far in the dementia disease …. And the idea that there's a disease is sometimes a new thing for these people. But an interesting [thing] is that, [they are] not seeking help, it's not only for people coming from the global south, but also people coming from Russia. There's also a kind of idea that it's a bit shame[ful] to have dementia… (Heidi) ‬‬‬‬‬‬‬‬‬‬‬‬‬‬‬‬‬‬‬‬‬‬‬‬‬‬‬‬‬‬‬‬‬‬‬‬‬‬‬‬‬‬‬‬‬‬

The professionals pointed out that hiding dementia is especially prevalent among people coming from Russia, Estonia, and Somalia, as well as among the Roma people. Consequently, we can argue that immigrants with dementia can experience more hardships compared with other persons living with dementia in general. This is because the tendency towards hiding dementia can restrict access to necessary care and support services, which can further compromise their health condition. Our professionals also pondered that it can be a daunting task to persuade people from other cultures to take a cognitive test for themselves or their relatives, as the dementia topic is usually avoided.

The professionals also mentioned that there is a misassumption among some religious immigrants; they consider dementia a part of God’s plan, and they cannot do anything about it. The professionals also pointed out that there is another common misassumption among some immigrants that dementia is considered a normal part of aging. This leads them to refrain from seeking help and support. One of the interviewees explained:

It starts a little bit earlier because they don't know even if dementia is [a] disease. This is a little bit difficult to go to get some services when I don't know that I need them and actually it's almost every group, every language group has that same kind of problem and we don't have terms to talk about dementia or memory disease or just getting old. A lot of … groups think that this is [a] normal part of aging. (Suvi, a Chief Executive Officer at an NGO)

Laura, who is a cultural services coordinator, explained that even a “doctor sometimes say[s] that, well, you are too old. This is like [a] normal thing. So they don’t suggest … checking” it out. Lack of knowledge about dementia and considering it a normal part of aging can have detrimental impacts on people’s health as they may not be able to receive proper diagnosis and treatment. This is why the provision of information may not only concern informing people about their rights, but it is also crucial to inform them about different diseases and treatments. The Finnish law requires the welfare areas to provide counselling and guidance to the population to promote their health and wellbeing as laid down in Section 6 of the Social Welfare Act, Section 12 of the Elder Care Act, and Section 13 of the Health Care Act, as well as identifying diseases, preventing, and treating them (The Health Care Act, Section 24). The Finnish laws in these cases can also work to empower older people and provide them with necessary knowledge to enhance their wellbeing, which can be argued to be line with Doron’s Empowerment Dimension*.* However, this may not always be guaranteed when some doctors do not offer older immigrants a cognitive test, which can further implant the myth about dementia as a normal part of aging among some immigrants. Dementia, however, is not a normal part of aging. It is a condition caused by a disease, such as Alzheimer’s disease (Kane and Thomas, 2017).

Cultural differences can be argued therefore to restrict the role of supportive family members in promoting the health of their older relatives. This contradicts the research findings about the “correlation between quality of life, health, and the ability to age in a respectful manner and the involvement of informal, social support networks” which Doron underlined in his Supportive Dimension (Doron, 2009, p. 65 cited Kane and Penrod, 1995). This is because family members can be viewed by their communities as supportive in some of these cases. For example, hiding dementia out of respect cannot be perceived as unsupportive. This can also apply to children who make decisions for their older parents. According to our professionals, family members believed that they were taking care of their relatives as they should according to their cultural norms. Thus, Doron’s Supportive Dimension does not take into account different cultural conceptions and their role in shaping different caring and support experience.

Nevertheless, in some cases, it was clear that family members were not supportive. Professionals pointed out that some parents seemed to be helped by their children in front of the authorities, but in reality, the parents would come asking for help, stating that their children were actually not helping them. In these cases, it can be difficult for us to assess whether the children pretended to help their older parents as a result of pressure from their communities to take care of their parents, or to receive the financial support given to them. This is because according to the Act on Support for Informal Care (937/2005, Section 2), family caregivers can receive a financial care allowance to support the provision of care to their family members. This is in line with Doron’s Supportive Dimension that stresses the importance of providing legal support to informal carers to promote the rights of their older relatives. However, cultural expectations and personal interests can undermine the role of carers, and in turn restrict the rights of immigrants.

### What Can Facilitate Access to Care and Support for Older Immigrants and Immigrants with Dementia?

According to the professionals, having someone to help was highly appreciated, especially regarding help with the language. Family and friends can also help with other obstacles related to the lack of digital skills. The professionals underlined that help from the immigrant community and NGOs is crucial especially for people who do not have anyone to help them. They highlighted the importance of the help provided by the communities and NGOs in reducing the pressure on service demands. Non-governmental organizations, however, have limited resources to address the increasing needs of different immigrant groups.

Nevertheless, the professionals stressed the need to establish services that can enable immigrants to seek adequate care and support directly from official authorities without reliance on others. This can be done by establishing a hotline service operated by the welfare area so that they can call and ask for help directly from the authorities in their native language. Shams, who works as an interpreter, suggested employing more bilingual staff so that immigrants can seek services directly from the service providers, as well as establishing care institutions for them.

According to our professionals, in addition, the provision of information in different languages about immigrants’ rights and available services is important to improving their lives. They also recommended other measures such as offering services to them as clients rather than immigrants, while respecting their cultural and religious backgrounds. The professionals stressed the importance of avoiding stereotypes and categorization, as this would enable service providers to provide the necessary services with less reluctance.

## Discussion

This study investigated the challenges that can face older immigrants and immigrants with dementia seeking care and support in Finland from the perspective of the professionals offering services to them. Our results showed an interconnectedness where one barrier to accessing care and support can lead to another. Limited skills in Finnish can restrict clients’ ability to use digital services and become acquainted with their rights. Combined with loneliness, these obstacles can increase the likelihood of abuse.

Like our study, previous research on older immigrants with Somali backgrounds living in Finland showed that the communication barrier between the doctors and the patients can restrict the patients’ ability to express their needs and leave them uncertain about the information they receive from their doctors (Mölsä and Tiilikainen, 2008). Moreover, a study conducted on Russian-speaking older immigrants in Finland highlighted the impact of limited digital skills, combined with poor Finnish language skills, on accessing digital services (Safarov, 2021).

Previous studies conducted abroad also highlighted the challenges associated with lacking language skills in accessing care and support services for older immigrants (Liu et al., 2017; Goettler, 2021; Yang et al., 2022; Li et al., 2018). The lack of linguistic skills additionally resulted in higher dependency on children (Polacsek and Angus, 2016) and in some cases increased the likelihood of abuse as a result of dependency on others (Guruge et al., 2021).

Nonetheless, on par with our results regarding immigrants with dementia, previous research from abroad also reported that the linguistic barrier negatively impacted the experiences of immigrants with dementia seeking care and support (Nielsen et al., 2020; Stevnsborg et al., 2016; Rosendahl et al., 2016; Söderman and Rosendahl, 2016; Sagbakken et al., 2018; Lee Casado et al., 2015; Kovaleva et al., 2021). Previous research, moreover, highlighted the link between lacking knowledge about dementia and the inability to seek treatment (Chaouni et al., 2020; Sun et al., 2014). Furthermore, lack of information about the available services and how to navigate the social and healthcare system also impeded immigrants’ access to care and support (Czapka and Sagbakken, 2020). The misunderstanding of dementia as part of normal aging (Stevnsborg et al., 2016; Cheung et al., 2019; Lee Casado et al., 2015) and as part of God’s plan (Czapka and Sagbakken, 2020) was also observed in previous studies.

However, according to this study and previous studies conducted in Finland (KC et al., 2023), the service system in Finland is built for native Finns, and presumptions and discrimination may exist in the Finnish service system. Previous research from abroad also underlines stereotypes and discrimination as barriers to access to services due to service systems developed for a homogenous society without considering the needs of ethnic minorities (Chaouni et al., 2020; Berdai Chaouni and De Donder, 2019).

Nevertheless, when it comes to cultural differences, the results from abroad also share similarities with the results from this study. For example, family members of immigrants with dementia showed a tendency to hide dementia from their relatives to make them feel that they are “functioning normally in daily life” (Vissenberg et al., 2018) or for the fear of hurting their feelings (Berdai Chaouni and De Donder, 2019). As a result, in both our study and previous studies abroad, relatives in these cases hindered their vulnerable family members from accessing help from service providers, consequently posing an obstacle to meeting their service needs. In some other cases, in contrast, informal social support networks helped the immigrant groups of our study address many of their challenges. If we take previous studies into account, having friends or someone to help also facilitated their access to care and support (Chung et al., 2018) and proved important in preventing loneliness (Liu et al., 2017).

As a lack of “cultural sensitivity and awareness” may hinder the provision of adequate social and medical services (Kankaanpää et al., 2023), previous studies have also recommended developing culturally sensitive services for ethnic minorities (Czapka and Sagbakken, 2020; Goettler, 2021), as well as more focus on employing bilingual staff (Sagbakken et al., 2020). Similarly to our study, previous research has also underlined the provision of information about available services as an important measure to improve the immigrant groups’ situation (Chung et al., 2018), as well as educating healthcare professionals on how to offer services to people from different cultural backgrounds (Vissenberg et al., 2018).

Nonetheless, previous research is limited when it comes to addressing legal aspects that can play an important role in the experiences of older immigrants and immigrants with dementia. This is why our study highlighted both the social and legal aspects as equally important to understand the phenomenon from a wider perspective. This provided a unique approach to analyzing our data and presenting our results. For example, we analyzed Finnish policies and highlighted issues that need to be addressed, such as the need to formulate policies that promote the capacity to use digital services. Our socio-legal approach also enabled us to identify a variety of factors that can impact the application of these laws and clash with them, and in turn, affect the experiences of older immigrants and immigrants with dementia, such as cultural differences, fear, and stereotypes.

Moreover, this empirical research involved Doron’s Multidimensional Model of Elder Law, a theoretical framework that was originally developed in the context of older people in general. Our study therefore uncovered the relevance of this theory within research that addresses the experiences of older immigrants and immigrants with dementia, extending as a result the perspective of this model. We also addressed the limitations in Doron’s model taking into account cultural differences and personal interests that may for instance conflict with the Supportive Dimension and the Preventive Dimension. In the case of Finland, we found out that many Finnish laws are in line with Doron’s dimensions, but some factors impacted the pertinence of some of these dimensions. Doron’s theoretical model could consequently benefit from incorporating more social aspects to address obstacles at the community level that can hinder older people’s enjoyment of their rights.

## Conclusions

This study investigated the obstacles that older immigrants and immigrants with dementia face when they seek care and support in Finland from the point of view of professionals offering services to them. Our results showed that limited Finnish skills, lack of digital skills, lack of information about rights, lack of expertise among service providers, loneliness, abuse, and cultural differences can restrict their access to justice.

Some of these challenges can be mitigated by developing culturally sensitive services in the immigrants’ native languages so that they seek help directly from the authorities. This can help them feel less reluctant to seek care and support. Culturally tailored practices can also help social and health care staff prepare adequate delivery of services to culturally diverse groups. The provision of information about the available services in different languages can also promote knowledge about their rights and how to seek them. Offering services to them like any other clients while respecting their cultural norms can help professionals avoid stereotypes and discrimination, as well as making them willing to take action when necessary. NGOs play an important role in facilitating the lives of older immigrants and immigrants with dementia and decreasing the pressure on the services provided by the public sector. However, their resources are limited, and thus it can be beneficial if state actors provided them with extra resources. Finally, employing more bilingual staff and educating social and healthcare professionals on how to deal with people from different backgrounds can make their job less challenging and in turn facilitate these immigrant groups’ access to justice.

### Strengths and Limitations of this Research

The study fills a gap in the research on the experiences of older immigrants and immigrants with dementia in Finland from both social and legal perspectives. While participants offered services to people from different countries, they all operated in the Southern Finland region. The provision of services and the experiences of older immigrants and immigrants with dementia might be different in other regions. Although we reached data saturation from the sample, four individual interviews lasted less than 30 minutes, which can be considered short in terms of providing comprehensive overview of the issues. Future research may involve conducting qualitative interviews with older immigrants and immigrants with dementia as it is crucial to study their firsthand experiences. This research, moreover, does not focus on issues related to discrimination, stigma, and self-sufficiency. Future research could involve these topics as they are deemed important to address.

## Data Availability

The data from the qualitative interviews that support the findings of this study will be publicly available from the Finnish Social Science Data Archive after the end of the project (31.08.2026).

## Declarations

**Ethical Approval**

Ethical approval for this study was received from the Committee on Research Ethics of the University of Eastern Finland (19/01/2023, Statement 4/2023). The research followed the guidelines set by the Finnish National Board on Research Integrity (Finnish National Board on Research Integrity TENK, 2019). All participants provided informed consent prior to their participation.
